# Correlates and inequality of underweight and overweight among women of reproductive age: Evidence from the 2016 Nepal Demographic Health Survey

**DOI:** 10.1371/journal.pone.0216644

**Published:** 2019-05-10

**Authors:** Anjana Rai, Swadesh Gurung, Subash Thapa, Naomi M. Saville

**Affiliations:** 1 Independent researcher, Kathmandu, Nepal; 2 Camris International, Kathmandu, Nepal; 3 Research Unit of General Practice, Department of Public Health, University of Southern Denmark, Odense, Denmark; 4 Visiting Researcher, Prince Naif Bin Abdulaziz Health Research Centre, King Saud University, Riyadh, Saudi Arabia; 5 Institute for Global Health, University College London, London, United Kingdom; University of Botswana, BOTSWANA

## Abstract

**Introduction:**

Understanding socio-economic correlates and inequality of underweight and overweight is crucial to develop interventions to prevent adverse health outcomes.

**Materials and methods:**

We analysed Nepal Demographic and Health Survey 2016 data from 6,069 women aged 15–49 years. WHO cut-offs for Body Mass Index categorised as: underweight (<18.5 kg/m^2^), normal weight (18.5kg/m^2^ to 24.9kg/m^2^) and overweight/ obesity (> = 25.0 kg/m^2^) were used. We used multinomial logistic regression to explore associations of factors with Body Mass Index and concentration indices to estimate socio-economic inequalities.

**Results:**

Higher risk of underweight was found in age group 15–19 (RRR 3.08, 95% CI: 2.29–4.15), 20–29 (RRR 1.64, 95% CI: 1.29–2.08) and in lowest (RRR 1.60, 95% CI: 1.03–2.47) and second wealth quintiles (RRR 1.77 (95% CI: 1.18–2.64). Education, occupation, urban/rural residence and food security were not associated with underweight (p>0.05). Lower risk of overweight/obesity was found in age group 15–19 (RRR 0.07, 95% CI: 0.05–0.10), 20–29 (RRR 0.40, 95% CI: 0.32–0.51), in manual occupation (RRR 0.58, 95% CI: 0.46–0.74) and in lower quintiles. Women with primary (RRR 1.91, 95% CI: 1.36–2.67), secondary education (RRR 1.42, 95% CI 1.00, 2.01) were at increased risk of overweight/obesity. Household food security and urban/rural residence were not associated with overweight/obesity (p>0.05). Socioeconomic inequalities were detected, with overweight/obesity strongly concentrated (concentration index: 0.380) amongst the higher quintiles and underweight concentrated (concentration index: -0.052) amongst the poorest.

**Conclusion:**

Nutrition programmes should target younger and poor women to address undernutrition and higher wealth group women to address overnutrition. Equity based nutrition interventions improving socio-economic status of poor households may benefit undernourished women. Interventions to encourage physical activity as women age and among wealthier women as well as healthy eating for prevention of under- and over-nutrition are needed.

## Introduction

The double burden of malnutrition is defined by the co-existence of serious levels of under- and overnutrition [1]. Under- and over-nutrition are major public health problems, as both are linked with increased risk of morbidity and mortality among women of reproductive age in low-income and middle-income countries [2]. In 2010, overweight and obesity accounted for a global 2·8 to 4·0 million deaths and 3·8% of Disability Adjusted Life Years [3]. Overweight and obesity lead to chronic illness, including increased risk of cardiovascular and metabolic diseases. For women of reproductive age, there are additional risks related to maternal health such as increased risk of cesarean section, complications during delivery and mortality [4–7]. Undernutrition, on the other hand, increases the risk of anemia and adverse maternal and child outcomes (e.g. neonatal death, stillbirths and low birth weight) [2,8–10]. South Asia had the highest prevalence of global underweight with 24% women being undernourished in 2014 [11].

In Nepal, prevalence of overweight and obesity is increasing (13% in 2011 to 22% in 2016) and underweight is decreasing (24% in 2006 to 17% in 2016), so a double burden of malnutrition among women of reproductive age exists in Nepal [12,13]. Moreover, the prevalence of underweight and overweight/obesity is higher among women than men [14]. According to the framework provided by Haddad et al., the determinants of both under- and overnutrition can be divided into three levels: immediate (e.g. health behaviours, biological factors), underlying (e.g. food environment, social environment) and basic (e.g. economic, political) [1]. These factors interact with each other to result in under- or over-nutrition. Rapid urbanization and increased income lead to increased access to high-calorie and processed foods and reduced physical activity, which in turn lead to increases in obesity and obesity-related chronic diseases [15,16]. More recently, overweight and its associated burden of non-communicable diseases (NCDs) has been recognized as a serious public, social and economic threat in Nepal and has been receiving an increasing amount of attention from researchers [17–19] and policy makers [20]. While public health programmes are already in place to address maternal micronutrient malnutrition in Nepal, such as the vitamin A and iron folate supplementation programmes, deworming and universal salt iodisation [21], undernutrition among women continues to cause high maternal and child mortality [22].

Inequalities in nutritional status across various socio-economic groups have been documented in several South Asian countries [23–27] and other low income- and middle income countries [24,28]. Generally overweight is more prevalent among the highest wealth quintiles while undernutrition is more prevalent amongst the lowest. Nutritional problems have been associated with socioeconomic factors, such as current work, possession of assets, urban or rural residence, household food security and demographic variables such as age, education, and marital status [6,11,24,29]. Several studies have assessed correlates of nutritional status among adults [14] and among women of reproductive age [13,27,30] in Nepal. However, associations of socioeconomic correlates and inequalities in nutritional status among women are not well described. Consequently, as the nutrition transition unfolds and trends in nutritional status continue to change, understanding factors associated with, and socioeconomic inequalities in, women’s under- and over-nutrition is important to guide the design and targeting of interventions.

Geoffrey Rose has posited that public health interventions targeting reduction of both under- and over-nutrition should work on shifting the risk distribution curve by a small amount in a population, and this approach has a greater beneficial effect in preventing morbidity and mortality than the interventions targeting only the individuals at higher risk [31]. Therefore, the question is, whether the “population” versus the “high risk” strategy is the optimal way to reduce the burden of under- and over-nutrition in Nepal. To our knowledge, no study from Nepal has utilized the latest nationally representative data to assess population characteristics associated with both underweight and overweight/obesity among the women of reproductive age in Nepal. Therefore, in this study, we examined the prevalence of underweight and overweight/obesity among women aged 15–49 years and its correlates using the recent 2016 Nepal Demographic and Health Survey (NDHS) data. Concentration indices have been popularly used to measure and compare the socioeconomic inequalities with many health-related variables including obesity status [32], therefore we used concentration indices to examine socio-economic inequalities in the distribution of underweight and overweight/obesity.

## Materials and methods

### Study design and setting

We used data from the nationally representative, cross-sectional NDHS, 2016 for this analysis. The NDHS survey is conducted every five years. In the 2016 NDHS, 383 wards (primary sampling units) were selected using probability proportional to ward size. Each sample was selected in two stages using stratified two-stage cluster sampling in rural areas and three-stage sampling in urban areas. 30 households per cluster were selected using an equal probability systematic selection method from the household listing (sampling frame). 11,473 households were finally selected for interview. The detailed methodology of the NDHS has been published elsewhere [12].

### Data sources

Of the total 12,862 women in the NDHS 2016, a sub-sample of 6069 women aged 15–49 years were interviewed and measured for anthropometry. The NDHS excludes pregnant women (n = 292) and women who had delivered within the last 2 months (n = 86) from anthropometric measurements. Ethical approval for the NDHS 2016 was obtained from Nepal Health Research Council and the participants provided written informed consent.

### Study Variables

#### Dependent variable

In the NDHS 2016, two female members and one male member of each team were trained on taking height and weight measurements and were standardized to check for accuracy and precision. Height and weight of women were measured just once. Height was measured to the nearest 0.1 cm using standard height boards and weight to the nearest 0.1 kg using digital weighing scales placed on a flat surface. Body mass index (BMI) was calculated by dividing weight (kg) by height (m^2^) and standard WHO cut-offs were used to determine underweight (<18.5 kg/m^2^), normal (> = 18.5 to 24.9 kg/m^2^), overweight/ obesity (> = 25.0 kg/m^2^) [33].

#### Independent variables

In our analysis, we included demographic and socio-economic covariates such as age, educational status, occupation, wealth quintile, household food security, residence and province. Women’s occupation was recoded into unemployed, non-manual and manual work using data on women’s employment in the 12 months before the survey. Professional, technical, managerial, clerical and sales/services were categorized as non-manual work; agriculture, skilled and unskilled manual were categorized as manual work. The NDHS 2016 collected information on household food security in the past 12 months using the nine-item household food insecurity access scale (HFIAS). The HFIAS questions arranged in order of severity and frequency of occurrence of households’ experience of food insecurity, were asked to household heads. For our analysis, we used data on the nine food security questions to calculate the four categories of food security: food secure, mildly insecure, moderately insecure and severely insecure using the HFIAS indicator guide [34]. For the measurement of socioeconomic status and inequality, we used wealth quintiles available in the NDHS 2016 dataset. These were constructed in the NDHS using principal component analysis of ownership of household assets, housing characteristics, source of drinking water and availability of toilet facilities and then divided into five equal quintiles [12].

### Statistical analyses

We weighted the dataset using the weights for primary sampling unit and checked for missing values and outliers before analysis. Descriptive statistics for women’s sociodemographic information were tabulated and disaggregated into underweight, normal weight, and overweight/obese categories. We performed univariate and multivariate multinomial logistic regression analyses to examine crude and adjusted associations between independent variables and underweight and overweight/obese nutritional categories of women compared with normal weight women. We chose the reference category for independent variables based on the least prevalence of underweight. The results are presented as relative risk ratios (crude and adjusted) with 95% confidence intervals. All statistical tests were adjusted for sampling design by applying sampling weights available in the NDHS 2016. A p value < 0.05 was considered statistically significant.

To assess socio-economic inequality in the distribution of underweight and overweight/obesity we used concentration curves and concentration indices. Two concentration curves were plotted; one for underweight and another for overweight/obesity. Concentration curves plot the cumulative percentage of underweight (or overweight/obese) in the y-axis against the cumulative percentage of the sampled women in the x-axis, ranked by wealth index from poorest to richest. They illustrate whether underweight (and overweight/obese) is more pronounced in the poorest or the richest.

The concentration index quantifies the socioeconomic inequality in underweight (or overweight/obesity) and is derived from the concentration curve. It estimates the area between the concentration curve and line of equality (45-degree angle), which is calculated as:

$$C=\frac{2}{\mu }COV\omega (yi,\;Ri)$$

where *yi* is the health status of the *i*^th^ individual and *Ri* is the fractional rank of the *i*^th^ individual in terms of the wealth index; *μ* is the weighted mean of *y*, and *COVω* is the weighted covariance.

The concentration index takes values between -1 and +1. When the values are negative, the curve lies above the line of equality, indicating that underweight or overweight/obesity is disproportionately concentrated among the poorer quintiles. When the values are positive, the curve lies below the line of equality, indicating that the underweight or overweight/obesity is disproportionately concentrated among the higher quintiles. If there is no socio-economic status-related inequality, the value is zero and the curve lies along the 45° line of equality [35]. All analyses were performed using STATA SE version 15.0.

## Results

After applying weights, a total of 6069 women aged 15–49 were available for analysis. Socio-demographic characteristics of women are given in Table 1. Mean (±SD) age of women was 29.5 years (± 9.8), one third had no education, around half were involved in manual work and 46.4% were food secure. Slightly more women (22.6%) belonged to fourth wealth quintile, more women were living in urban area (63.2%) and were from province 3 (22.3%).

**Table 1:** Socio-demographic characteristics of women in the study (N = 6069).

Variables	Categories	Mean	SD
**Age**		29.5	9.8
		**%**	**95% CI**
**Education**	None	33.9	[32.0–35.9]
	Primary	16.3	[15.0–17.8]
	Secondary	35.5	[33.8–37.2]
	Higher	14.3	[12.9–15.8]
**Occupation**	Not working	31.6	[29.2–34.1]
	Non-manual	15.3	[13.6–17.2]
	Manual	53.1	[50.1–56.1]
**Wealth quintile**	Lowest	17.0	[14.8–19.4]
	Second	18.9	[17.0–20.9]
	Middle	20.3	[18.4–22.2]
	Fourth	22.6	[19.8–25.7]
	Highest	21.3	[18.5–24.3]
**Food security**	Food secure	46.4	[43.7–49.1]
	Mildly insecure	23.7	[21.9–25.6]
	Moderately insecure	22.2	[20.4–24.1]
	Severely insecure	7.7	[6.6–9.0]
**Residence**	Urban	63.2	[58.6–67.5]
	Rural	36.8	[32.5–41.4]
**Province**	Province 1 (eastern region)	16.9	[15.6–18.4]
	Province 2 (central / eastern plains)	19.3	[17.6–21.1]
	Province 3 (central including Kathmandu)	22.3	[18.6–26.5]
	Province 4 (western region)	10	[8.9–11.1]
	Province 5 (western/mid-western region)	17	[15.5–18.5]
	Province 6 (Karnali region)	5.7	[5.2–6.3]
	Province 7 (far-western region)	8.8	[7.8–9.9]

**Fig 1:**
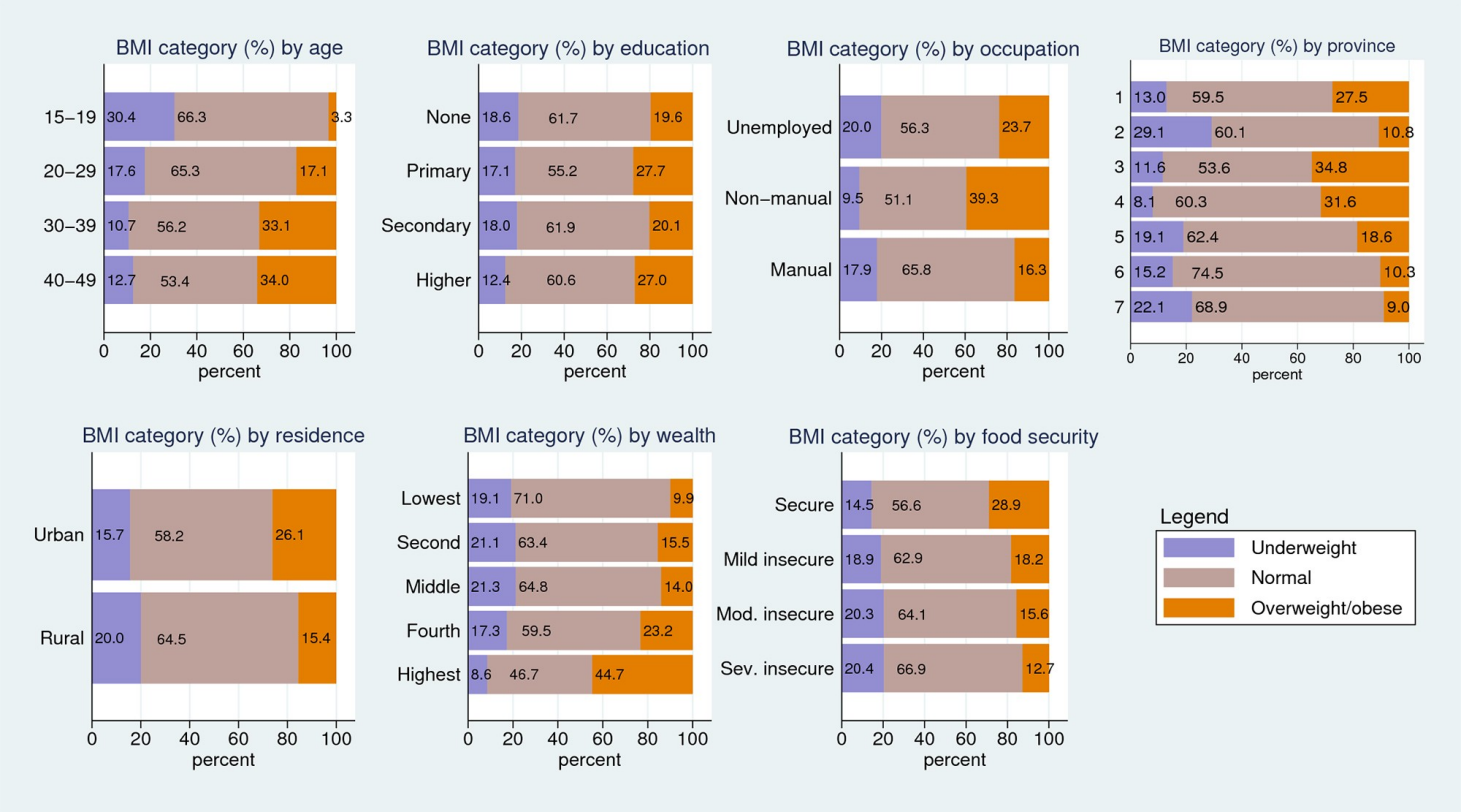
Prevalence of underweight, normal weight and overweight/ obese by socio-demographic characteristics (%) (N = 6069).

The overall prevalence of underweight was 17.3% (95% CI: 15.8–18.8) and of overweight was 22.3% (95% CI: 20.4–24.0) respectively. Fig 1 illustrates the distribution of underweight, normal weight and overweight/obesity by age, education, occupation, wealth quintile, food security, residence and province. Underweight was most prevalent in the 15–19 age group (30.4%, 95% CI: 27.5–33.3), among women who had no education (18.6%, 95% CI: 16.5–21.0), were unemployed (20%, 95% CI: 17.8–22.4), in the second and middle wealth quintiles (~21%), in moderately and severely food insecure households (~20%), in rural areas (20.0%, 95% CI: 17.6–22.8) and in province 2 (29.1%, 95% CI: 25.7–32.8) followed by province 7 (22.1%, 95% CI: 18.5–26.2).

Overweight/obesity was most prevalent in the 40–49 age group (34.0%, 95% CI: 30.1–38.1), amongst women with higher education and primary education (~27%), in non-manual occupation (39.3%, 95% CI: 34.6–44.3), in highest wealth quintile (44.7%, 95% CI: 40.9–48.6), in food secure households (28.9%, 95% CI: 26.3–31.6), urban areas (26.1%, 95% CI: 23.5–28.8) and in province 3 (34.8%, 95% CI: 29.5–40.6).

### Correlates of underweight and overweight/obesity

Table 2 shows relative risk ratios of crude and adjusted multinomial logistic regression models for underweight and overweight/obesity relative to normal weight women and associations with both. The crude regression models showed significantly higher risk of being underweight in the following: women aged 15–19 and 20–29 years relative to women 30–39 years; women without higher secondary education relative to women educated to higher secondary or above; women who were unemployed or in manual occupation relative to those in non-manual employment; those in the 4 lower wealth quintiles relative to the highest; from moderately food insecure households compared with food secure households. Compared to women in province 4, women in all other provinces had significantly increased risk of being underweight. However, living in urban or rural area was not associated with being underweight compared to normal weight women.

**Table 2:** Crude and adjusted relative risk ratios for correlates of underweight and overweight/obese in comparison with normal weight women (6069).

Possible correlates of nutritional status	Crude		Adjusted	
	Underweight	Overweight/obese	Underweight	Overweight/obese
	RRR (95% CI)	RRR (95% CI)	RRR (95% CI)	RRR (95% CI)
**Age**				
15–19	2.40** (1.88–3.05)	0.08** (0.06–0.13)	3.08** (2.29–4.15)	0.07** (0.05–0.10)
20–29	1.41* (1.15–1.74)	0.44** (0.37–0.54)	1.64** (1.29–2.08)	0.40** (0.32–0.51)
30–39	**Ref**.			
40–49	1.24 (0.94–1.64)	1.08 (0.89–1.31)	1.21 (0.92–1.61)	1.17 (0.93–1.46)
**Education**				
No education	1.48* (1.12–1.97)	0.72* (0.54–0.94)	1.42 (0.99–2.03)	1.23 (0.83–1.83)
Primary	1.52* (1.11–2.09)	1.13 (0.89–1.43)	1.33 (0.94–1.88)	1.91** (1.36–2.67)
Secondary	1.43* (1.09–1.86)	0.73* (0.56–0.96)	1.02 (0.77–1.36)	1.42* (1.00–2.01)
Higher secondary	**Ref**.			
**Occupation**				
Unemployed	1.90** (1.39–2.59)	0.55** (0.43–0.69)	1.18 (0.88–1.59)	0.95 (0.75–1.20)
Non-manual	**Ref**.			
Manual	1.46* (1.11–1.93)	0.32** (0.26–0.40)	0.99 (0.73–1.33)	0.58** (0.46–0.74)
**Wealth quintile**				
Lowest	1.46* (1.09–1.96)	0.15** (0.11–0.19)	1.60* (1.03–2.47)	0.22** (0.15–0.31)
Second	1.81** (1.33–2.47)	0.26** (0.20–0.32)	1.77* (1.18–2.64)	0.35** (0.27–0.46)
Middle	1.79** (1.32–2.42)	0.23** (0.17–0.29)	1.40 (0.99–2.05)	0.33** (0.25–0.44)
Fourth	1.58* (1.17–2.12)	0.41** (0.33–0.50)	1.35 (0.99–1.84)	0.51** (0.40–0.65)
Highest	**Ref**.			
**Household Food security**				
Food secure	**Ref**.			
Mildly insecure	1.18 (0.96–1.46)	0.57** (0.46–0.71)	0.96 (0.77–1.20)	0.90 (0.72–1.14)
Moderately insecure	1.24* (1.02–1.52)	0.48** (0.38–0.59)	1.10 (0.88–1.38)	0.79 (0.62–1.01)
Severely insecure	1.19 (0.87–1.64)	0.37** (0.26–0.54)	1.02 (0.72–1.46)	0.69 (0.47–1.03)
**Place of residence**				
Urban	**Ref**.			
Rural	1.15 (0.93–1.43)	0.53** (0.43–0.66)	0.98 (0.78–1.22)	1.01 (0.83–1.23)
**Province**				
1 (eastern region)	1.63* (1.02–2.60)	0.88 (0.67–1.16)	1.60* (1.01–2.54)	0.87 (0.67–1.15)
2 (central / eastern plains)	3.62** (2.58–5.09)	0.34** (0.25–0.46)	3.56** (2.49–5.09)	0.29** (0.22–0.39)
3 (central including Kathmandu)	1.62* (1.02–2.57)	1.24 (0.92–1.67)	1.70* (1.07–2.69)	0.94 (0.74–1.19)
4 (western region)	**Ref**.			
5 (western/mid-western region)	2.28** (1.60–3.25)	0.57** (0.43–0.75)	2.31** (1.63–3.28)	0.52** (0.39–0.67)
6 (Karnali region)	1.53* (1.03–2.27)	0.26** (0.18–0.39)	1.35 (0.90–2.03)	0.42** (0.29–0.60)
7 (far-western region)	2.39** (1.69–3.40)	0.25** (0.15–0.42)	2.34** (1.65–3.30)	0.30** (0.19–0.46)

*p<0.05

**p<0.001

Crude regressions showed significantly lower risk of overweight/obesity in the following: women aged 15–19 and 20–29 years relative to 30–39 years; women with who had no education or secondary education relative to higher secondary or above; women who were unemployed or had manual occupation relative to those in non-manual employment; women in the lower 4 wealth quintiles relative to the highest wealth quintile; women who experienced food insecurity relative to food secure; women in rural areas compared with urban; and women living in Provinces 1, 2, 5 and 7 relative to Province 4.

When the multivariate multinomial logistic regression model was mutually adjusted for age, education, occupation, wealth, food insecurity, residence and province, a number of changes were found. The relative risk of being underweight increased from 2.40 (95% CI: 1.88–3.05) to 3.08 (95% CI: 2.29–4.15) for 15–19 age group and from 1.41 (95% CI: 1.15–1.74) to 1.64 (95% CI: 1.29–2.08) for 20–29 age group when compared to women in age group 30–39. In the adjusted model, women from lowest (RRR 1.60, 95% CI: 1.03–2.47)) and second (RRR 1.77, 95% CI: 1.18–2.64)) wealth quintiles were at increased risk of being underweight relative to the highest, but the association with other wealth quintiles, education, occupation and food security disappeared. After adjustment, women in Province 2 were at 3.56 times (95% CI: 2.49–5.09) increased risk of being underweight and women in Provinces 1, 3, 5, 7 had approximately doubled risk of being underweight compared to women in Province 4.

In adjusted models, associations with overweight/obesity also disappeared for women with no education relative to higher secondary and above, for women who were unemployed relative to non-manual labour and for those living in rural areas. Associations between overweight/obesity and age remained similar. Younger women aged 15–19 years (RRR 0.07, 95% CI: 0.05–0.10) and women aged 20–29 years (RRR 0.40, 95% CI: 0.32–0.51) had significantly lower risk of being overweight/obese compared to women aged 30–39 years. Women with primary education were at doubled risk (RRR 1.91, 95% CI: 1.36–2.67) and women with secondary education were at 1.42 times (95% CI: 1.00–2.01) increased risk of being overweight/obese relative to those with higher secondary education and above. Women who were involved in manual occupation (RRR 0.58; 95% CI: 0.46–0.74) compared to women involved in non-manual occupation and women from all quintiles compared to highest quintile households had significantly lower risk of being overweight/obese and the size of the RRR increased with adjustment for wealth. Women from province 2 (RRR 0.29, 95% CI: 0.22–0.39), province 5 (RRR 0.52, 95% CI: 0.39–0.67), province 6 (RRR 0.42, 95% CI: 0.29–0.60) and province 7 (RRR 0.30, 95% CI: 0.19–0.46) had significantly lower risk of being overweight/ obese compared to women from Province 4.

The overall concentration index for underweight was -0.052 (SE: 0.025; *p* = 0.043). This small negative value indicates that the poorest households bear the highest burden of underweight. This confirms the significantly higher relative risk of underweight in the four lower wealth quintiles compared with the highest (Table 2). However, the concentration index value is small and should be interpreted with caution.

**Fig 2:**
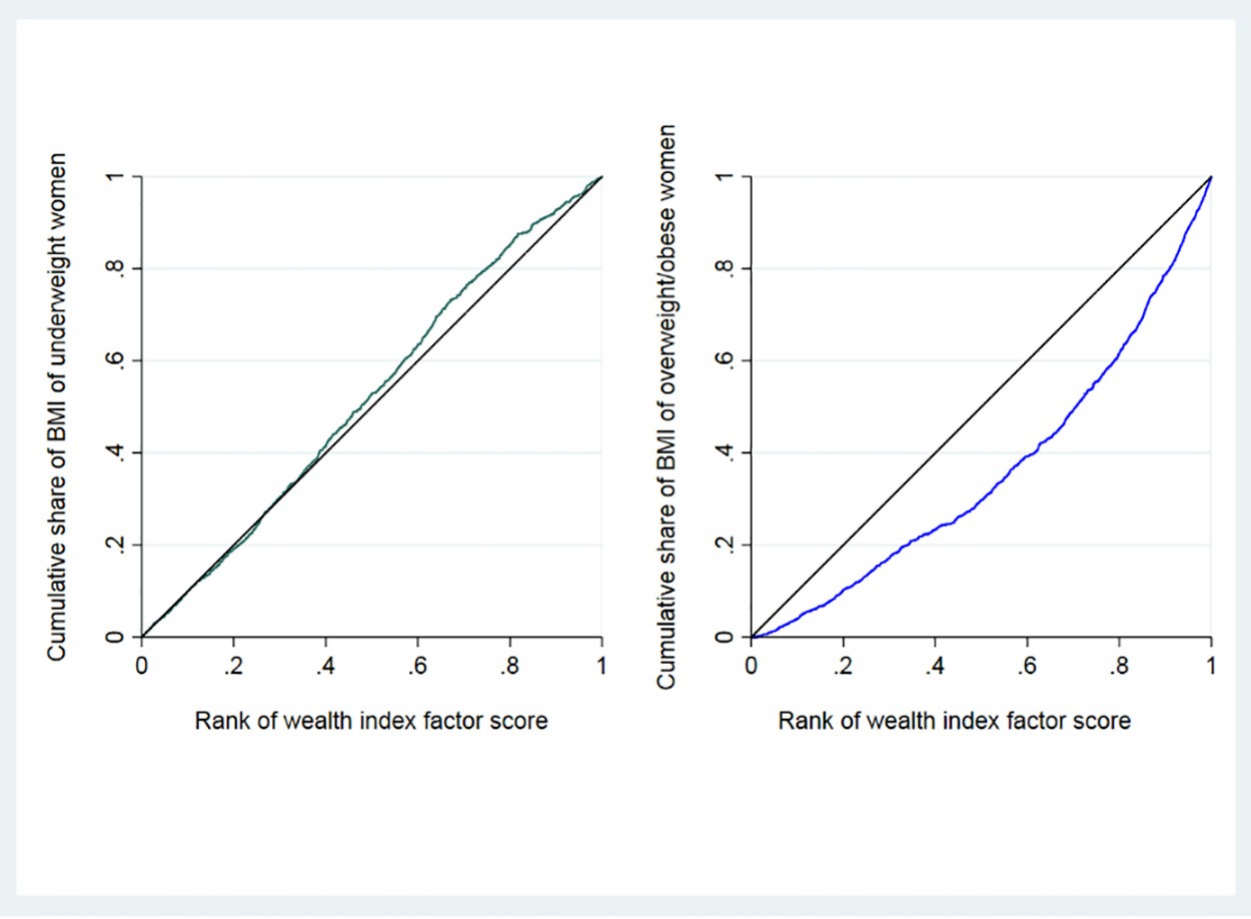
Concentration curves for underweight and overweight/obese.

The concentration index for overweight/obese was 0.380 (SE: 0.025; *p*<0.001) indicating that the higher wealth quintiles bear the burden of overweight/obesity (Fig 2), as also shown the significantly lower adjusted risk ratios for overweight and obesity relative to the highest wealth quintile in Table 2.

Considering that other readers may be interested in use of Asian BMI cut-offs; we have provided a supplementary table (S1 Table) on multinomial logistic regression (crude and adjusted) using the Asian BMI cut-offs. Even though the direction of association is similar to that seen in adjusted model using international BMI cut-offs (Table 2), in the adjusted model using Asian cut-offs, no education and primary education were significantly associated with increased risk of being underweight while moderately food insecure and province 1 was significantly associated with reduced risk of being overweight/obese. However, the association of lowest wealth quintile and province 1 with being underweight as observed in the model using international BMI cut-offs, was not significant in the adjusted Asian BMI cut-off model (S1 Table). The associations of age, occupation and residence with underweight and overweight/obese using the Asian BMI cut-off are similar to the associations observed using international BMI cut-offs (Table 2).

## Discussion

We assessed the relationship between socio-economic variables and the risk of both under-nutrition and overweight/obesity and assessed socio-economic inequalities in both under-nutrition and overweight/obesity among women of reproductive age in Nepal. We found that age of the women and wealth were most strongly associated with both under- and over-weight after adjustment for education, food security, rural versus urban residence, occupation and province. The disparities in prevalence of underweight and overweight/obese by wealth quintiles, as well as increased risk of overweight and decreased risk of underweight with increasing wealth found in our study is consistent with other studies conducted in low income and middle-income countries [25–28,36]. The increase in prevalence of overweight/obesity in Nepal over the last decade is also especially noticeable in highest quintile [12,37,38]. Moreover, problems of underweight and overweight/obesity among women are inequitably distributed across socio-economic quintiles, with concentration of underweight among the poorer and overweight/obesity among the richer. The concentration index that we found for overweight/obese (0.38, p<0.001) was close to that found for Bangladesh (0.45) [39]. Similar socio-economic inequality was observed in a multi-national Sub-Saharan African study which reported a concentration index of 0.23 (p<0.001) for overweight/obesity indicating its concentration among the higher socioeconomic groups, compared to the lower [40].

Bhandari et al. (2016) found increased risk of underweight and decreased risk of overweight/obesity among younger women in Nepal with women aged 15–24 years 2.7 times more likely to be underweight [41]. Kamal et al. (2015) in Bangladesh similarly found that younger women were more likely to be underweight and less likely to be overweight/obese [23], and other studies have also reported age to be significantly associated with BMI [14,42]. These findings should alert program planners to develop nutrition programmes for young reproductive aged women and adolescents to prevent the adverse consequences of poor preconception nutrition.

We might expect that women with better education have better nutrition knowledge to make better-informed decisions about their diet. However, we found after adjusting for wealth, that women with primary or secondary education had a higher risk of being overweight/obese. This relationship could be partially driven by the fact that educated women may be in less physically demanding jobs and that even educated people may be unaware of the health risks of being overweight. Our finding is in contrast with a recent study from Nepal among male and female adults using 2016 NDHS data [14] and a study of women of reproductive age using 2011 NDHS data [30], which did not find any association of primary or secondary education with being overweight/obese. Comparing NDHS data for 2011 and 2016 shows that mean BMI and percentage overweight/obese increased markedly in all education groups. Whilst the percentage overweight/obese for uneducated women increased from 10.8% to 19.6%, for primary and secondary educated women, it increased from 15.5 to 27.7% and from 12.7 to 20.8% overweight/obese respectively. The larger increase in overweight/obesity in the primary and secondary educated women explains the difference between our study and the 2011 study by Singh et al [30]. The difference between Rawal et al’s analysis of NDHS 2016 data and our own is explained by the fact that their analysis included men whose BMI is not associated with education, whilst our analysis included reproductive age women only.

We found women involved in manual work had a lower risk of being overweight/obese than those in non-manual work. This may be due to higher levels of physical activity which have been shown to promote weight loss [43,44].

Absence of association between food insecurity and underweight was contrary to the findings from other studies [30,45,46] who found that food insecurity was associated with undernutrition. This is expected as food security is usually associated with higher dietary diversity, meal frequency and amount of food consumed. Food security was a not associated with overweight/obesity in our study nor in studies in Bangladesh [23] and Colombia [46]. We speculate that, in our study, food security may have affected nutritional status through income since the association with moderate food insecurity and underweight disappears when wealth is adjusted for. This is consistent with the findings of others who found food security to be associated with income [47,48]. This suggests that the government of Nepal and its development partners need to concentrate on promotion of nutrition sensitive activities that address poverty by promoting income generating activities such as agriculture. This is consistent with Osei et al. (2016) who found promotion of homestead food production in Nepal to be effective in reducing food insecurity and improving maternal underweight and maternal and child anemia [49].

We did not observe any rural and urban differences in the adjusted multivariate model for either underweight or overweight/obesity in our study as the rural/urban differences were explained by differences in wealth, occupation and education in rural and urban areas. It might also be an indication of a nutrition transition where underweight and overweight/obesity is prevalent across all food security groups and in both urban and rural locations. Further studies should look at pathways that influence food security and its effect upon nutritional status as well as diet patterns in urban and rural areas which might help explain the findings seen in this study.

We found a significant difference in nutritional status between the seven recently formed federal administrative provinces in Nepal, suggesting the need for context-specific nutrition interventions in different regions of the country. Women from provinces 2, 5 and 7 had increased risk of being underweight and decreased risk of being overweight relative to province 4. In the case of Province 2 this may be because of low dietary diversity among women owing to low intakes of fruits, vegetables and animal foods [50,51], whereas in the remote hilly and mountain districts of provinces 5 and 7 food insecurity might be the main cause [49]. There might also be inequitable intrahousehold allocation of food which might result in low food intake, especially in Province 2 [52,53]. It is surprising that compared with Province 4, women from Province 6 were not at increased risk of being underweight, since this Province has the highest levels of food insecurity [12]. Further research is needed to understand the drivers of under- and over-weight in this part of Nepal. Despite the highest prevalence of overweight/obesity (35%) in Province 3 where the Kathmandu valley lies, we attribute the increased risk of underweight in Province 3 to inadequate dietary intake and multiple infections [54,55]. A recent study conducted in the Kathmandu valley reported that 76% of participants consumed fast food and 39% had a sedentary lifestyle in the preceding week [56]. This could explain the high prevalence of obesity in Province 3. More studies exploring these differences between provinces are needed.

Studies from Nepal have suggested that rapid urbanization, dietary changes towards processed foods and consumption of more starchy food (e.g. rice and potatoes) and less vegetables and fruits, could be the reasons for the increasing burden of overweight/obesity in Nepal [41,57]. Moreover, Nepalese livelihoods used to be based on traditional farming and animal husbandry but are now largely based upon labour migration and remittances. As a result, in recent years, there has been significant reduction in the production and consumption of traditional crops (e.g. millet, green vegetables, maize, pulses etc.) and concurrently an increase in consumption of white rice, oil and sugar and sedentary lifestyles [57]. This rise in overweight and obesity associated with these socio-cultural factors indicates the continued importance of integrating NCD prevention in national health services.

Our findings around risk factors for under- and over-nutrition in Nepal suggest that multilevel interventions are needed that target people of differing income groups and population sub-groups (e.g. uneducated and working women; women from different age groups). Our study shows that wealth is clearly linked with the distribution of under- and over-weight, and thus poverty reduction interventions to help women to have their own income and increase the production and consumption of green vegetables and fruits (e.g. microfinance, kitchen gardening, and small businesses) could have some promising results [58–60]. At the community level, women’s group initiatives which have been evidenced to improve nutrition knowledge and maternal and neonatal health [61,62] could be an important strategy to promote physical activity and respond to the double burden of nutritional problems in both rural and urban areas. Additionally, social and behavior change communication covering a wide population to increase awareness about healthy diet, nutrition and lifestyle may be beneficial [63,64]. At an individual level, increased intake of micronutrient-rich fruits and vegetables could benefit both underweight and overweight women whilst increased intake of animal foods and more equitable intra-household food allocation could benefit underweight women [52]. In addition, under-weight women should be properly screened and treated for infections, anemia and micronutrient deficiency disorders; whereas over-weight women should be screened regularly for risks of non-communicable diseases.

### Limitations of the study

Our study has some limitations. First, due to the cross-sectional nature of the DHS survey, we cannot infer causality of independent variables upon nutritional status. Second, because of limited number of variables available to us, the analysis did not include all probable correlates, particularly the variables related to dietary diversity or other aspects of dietary intake such as intra-household food allocation, which are associated with nutritional status among women. Future research should further investigate the effect of dietary diversity and intra-household food allocation on women’s nutritional status in Nepal. Third, we had to use an asset-based wealth index as a proxy of socio-economic status, as the NDHS does not collect information on household income and expenditure. Fourth, the NDHS does not include adolescent girls below 15 years of age, which makes it difficult to draw conclusions about adolescents based on the 15–19 groups. Despite these limitations, the analysis and results presented in this study are based on a nationally representative survey, which provides strong evidence of potential correlates of nutritional status of women of reproductive age in Nepal.

## Conclusion

In Nepal, age and wealth quintiles are most strongly associated with both under- and over nutrition. Underweight is concentrated amongst young, poorer women whilst overweight and obesity is concentrated in wealthier subgroups. Development of equity-based health and nutrition programmes are needed whilst improving the socio-economic status of the poor in order to see major reductions in undernutrition in Nepal; especially targeting adolescents and young women. Tailored interventions for prevention of overweight/obesity as women age and in the higher wealth quintiles are also urgent, but nutrition education for all would also be beneficial. In the new federal system, local governments should develop nutrition specific and nutrition sensitive interventions to mobilize local resources for addressing the multi-dimensional issues that pre-dispose women to be under and over-nourished.

## Supporting information

S1 Table Crude and adjusted relative risk ratios for correlates of underweight and overweight/obese in comparison with normal weight women (6069) using Asian BMI-cutoffs. (PDF)
