# Supplementary material for: Correlates and inequality of underweight and overweight among women of reproductive age: Evidence from the 2016 Nepal Demographic Health Survey
Source: PLoS One. 2019 May 10;14(5):e0216644. doi: 10.1371/journal.pone.0216644 (PMC6510472; doi:10.1371/journal.pone.0216644)
Supplement: S1 Table — (PDF) [file pone.0216644.s001.pdf]

S1 Table: Crude and adjusted relative risk ratios for correlates of underweight and overweight/obese in comparison with normal weight women (6069) using Asian BMI-cutoffs

Possible correlates of nutritional status	Crude		Adjusted	
	Underweight	Overweight/obese	Underweight	Overweight/obese
	RRR (95% CI)	RRR (95% CI)	RRR (95% CI)	RRR (95% CI)
**Age**				
15-19	2.06** (1.61 - 2.64)	0.19** (0.15 - 0.24)	2.56** (1.51 - 2.68)	0.16** (0.12 - 0.20)
20-29	1.32* (1.07 - 1.64)	0.52** (0.45 - 0.60)	1.51** (0.90 - 1.57)	0.48** (0.40 - 0.56)
30-39	**Ref.**			
40-49	1.29 (0.98 – 1.70)	1.13 (0.94 – 1.34)	1.27 (0.96 – 1.68)	1.21 (1.00 – 1.47)
**Education**				
No education	1.50* (1.12 - 2.00)	0.82 (0.64 - 1.06)	1.47* (1.01 - 2.13)	1.22 (0.87 - 1.72)
Primary	1.65* (1.20 - 2.26)	1.28* (1.00 - 1.63)	1.49* (1.06 - 2.11)	1.96** (1.38 - 2.78)
Secondary	1.43* (1.09 - 1.86)	0.82 (0.63 - 1.06)	1.11 (0.83 - 1.47)	1.51* (1.06 - 2.15)
Higher secondary	**Ref.**			
**Occupation**				
Unemployed	1.77** (1.29 - 2.42)	0.56** (0.46 - 0.69)	1.17 (0.86 - 1.58)	0.93 (0.76 - 1.15)
Non-manual	**Ref.**			
Manual	1.36* (1.03 - 1.80)	0.41** (0.34 - 0.50)	0.96 (0.71 - 1.30)	0.68** (0.55 - 0.85)
**Wealth quintile**				
Lowest	1.30 (0.96 - 1.76)	0.24** (0.19 - 0.30)	1.47 (0.93 - 2.30)	0.35** (0.25 - 0.48)
Second	1.63* (1.19 - 2.25)	0.33** (0.26 - 0.41)	1.64* (1.07 - 2.51)	0.45** (0.34 - 0.61)
Middle	1.60* (1.16 - 2.22)	0.30** (0.24 - 0.37)	1.32 (0.87 - 1.99)	0.46** (0.35 - 0.60)
Fourth	1.45* (1.05 - 1.98)	0.45** (0.36 - 0.56)	1.26 (0.90 - 1.77)	0.57** (0.44 - 0.73)
Highest	**Ref.**			
**Household Food security**				
Food secure	**Ref.**			
Mildly insecure	1.09 (0.88 - 1.35)	0.58** (0.48 - 0.71)	0.92 (0.73 - 1.15)	0.82 (0.66 - 1.01)
Moderately insecure	1.12 (0.91 - 1.37)	0.48** (0.41 - 0.58)	1.02 (0.81 - 1.28)	0.69** (0.56 - 0.83)
Severely insecure	1.17 (0.88 - 1.55)	0.54** (0.38 - 0.77)	1.02 (0.74 - 1.40)	0.86 (0.57 - 1.28)
**Place of residence**				
Urban	**Ref.**			
Rural	1.10 (0.89 - 1.36)	0.60** (0.51 - 0.72)	0.97 (0.77 - 1.21)	0.97 (0.81 - 1.15)
**Province**				
1 (eastern region)	1.48 (0.94 - 2.34)	0.76* (0.59 - 0.98)	1.46 (0.93 - 2.30)	0.74* (0.58 - 0.95)
2 (central / eastern plains)	3.08** (2.21 - 4.28)	0.35** (0.27 - 0.45)	3.01** (2.12 - 4.27)	0.31** (0.25 - 0.40)
3 (central including Kathmandu)	1.64* (1.02 - 2.63)	1.17 (0.94 - 1.47)	1.70* (1.05 - 2.73)	0.97 (0.79 - 1.19)
4 (western region)	**Ref.**			
5 (western/mid-western region)	2.14** (1.52 - 3.01)	0.62** (0.49 - 0.79)	2.15** (1.53 - 3.02)	0.59** (0.47 - 0.73)
6 (Karnali region)	1.34 (0.91 - 1.96)	0.34** (0.25 - 0.46)	1.23 (0.82 - 1.82)	0.49** (0.37 - 0.65)
7 (far-western region)	2.04** (1.45 - 2.87)	0.30** (0.21 - 0.43)	2.03** (1.44 - 2.85)	0.35** (0.26 - 0.48)

*p<0.05 **p<0.001
